# Tenofovir Disoproxil Fumarate Is Associated with a Set-Point Variation in the Calcium-Parathyroid Hormone-Vitamin D Axis: Results from a German Cohort

**DOI:** 10.1155/2018/6069131

**Published:** 2018-12-31

**Authors:** Sebastian Noe, Silke Heldwein, Carmen Wiese, Rita Pascucci, Ariane von Krosigk, Farhad Schabaz, Celia Jonsson-Oldenbuettel, Hans Jaeger, Eva Wolf

**Affiliations:** ^1^MVZ Karlsplatz, Research and Clinical Care Center, 80335 München, Germany; ^2^MUC Research, 80335 München, Germany

## Abstract

**Background:**

Higher levels of parathyroid hormone have been associated with the use of tenofovir disoproxil fumarate (TDF) in people with and without HIV infection. Yet, alterations in calcium levels have never been elucidated in detail.

**Objective:**

To compare the association of parathyroid hormone with serum calcium levels and other markers of calcium and bone metabolism in people living with HIV on TDF- and non-TDF-containing antiretroviral therapy.

**Patients and Methods:**

A retrospective single center cohort study in Munich, Germany. Median and interquartile ranges and absolute and relative frequencies were used to describe continuous and categorical variables, respectively. The Mann–Whitney *U* test and chi^2^-test were used for comparisons. Multivariate median regression was performed in a stepwise backward approach.

**Results:**

1,002 patients were included (786 (78.4%) male; median age 48 (40–55) years). 564 patients (56.3%) had a TDF-containing ART regimen. PTH concentrations were 46.9 (33.0–64.7) pg/mL and 35.2 (26.4–55.4) pg/mL (*P*=0.001), 43.3 (30.8–59.8) pg/mL and 31.8 (22.3–49.6) pg/mL (*P* < 0.001), 46.1 (29.5–65.4) pg/mL and 33.4 (22.6–50.1) pg/mL (*P* < 0.001), and 37.8 (25.3–57.9) pg/mL and 33.8 (20.1–45.3) pg/mL (*P*=0.012) within the first, second, third, and fourth quartile of corrected calcium levels for patients with and without TDF-containing ART, respectively. In multivariate median regression, PTH concentration was significantly associated with Ca_corr._ (−32.2 (−49.8 to −14.8); *P* < 0.001), female sex (5.2 (1.2–9.2); *P*=0.010), 25(OH)D (−0.4 (−0.5 to −0.3); *P* < 0.001), and TDF-use (9.2 (6.0–12.5); *P* < 0.001).

**Discussion:**

Higher levels of PTH seem to be needed to maintain normal calcium levels in PLWH on TDF-containing ART compared to non-TDF-containing ART. Optimal concentrations for 25-hydroxy vitamin D and calcium might therefore be different in people using TDF than expected from general populations but also people living with HIV with non-TDF-containing antiretroviral therapy. This might require different supplementation strategies but warrants further investigation.

## 1. Introduction

Tenofovir disoproxil fumarate (TDF) has been associated with increased levels of parathyroid hormone (PTH) in both, people living with (PWLH) and without HIV [1–4]. The underlying mechanisms have, however, been poorly understood until recent preclinical data demonstrated a direct inhibitory effect of TDF on the calcium-sensing receptor (CaSR), leading to inhibition of the negative feedback regulation of calcium on PTH secretion and therefore higher levels of PTH [5], translating into a change in the serum calcium set-point. But, the pharmacokinetics of any drug that interacts with the CaSR is as important as the interaction itself and will determine the overall effect, particularly on bone: while intermittent elevations of PTH might have the potential to increase bone mass, continuous elevated levels of PTH (e.g., found in primary hyperparathyroidism) lead to an increased risk of osteoporosis.

As tenofovir concentrations demonstrate a peak after oral intake of TDF but remain elevated between two dosing intervals [6], TDF might lead to continuously rather than intermittently elevated PTH levels and might explain why TDF has been associated with osteoporosis rather than its treatment. A better understanding of the interference of TDF with calcium homeostasis seems, however, of impact on care of people using TDF, including PLWH, patients with chronic hepatitis B, as well as the increasing number of people taking TDF for pre-exposure prophylaxis (PrEP). An altered serum calcium set-point—as indicated by the aforementioned study [5]—might lead to different requirements for people using TDF compared to general populations or PLWH on non-TDF-containing antiretroviral therapy (ART), particularly with regard to calcium and vitamin D. It might furthermore help guiding us in the decision about which subgroup of TDF-users should be discontinued from TDF and switched to alternative medications, including the TDF-successor tenofovir alafenamide (TAF).

We therefore aimed to describe the relation between PTH and (albumin-corrected) serum calcium levels as well as levels of 25-hydroxy vitamin D (25(OH)D) in PLWH with and without TDF-containing ART. We hypothesized that PTH levels in TDF-treated patients will be higher for given albumin-corrected serum calcium levels (Ca_corr._) throughout the range of Ca_corr._. We furthermore aimed at exploring the effect of 25(OH)D levels on PTH suppression in PLWH with and without TDF-containing ART, hypothesizing that higher levels of 25(OH)D will be needed to achieve comparable levels of PTH in patients on TDF versus non-TDF-containing ART.

## 2. Patients and Methods

Data derived from a cohort of a single HIV clinical care and research center in Munich, Germany, have in part been published before [3]. Patients who attended the clinic in 2016 were considered for enrolment, if calcium, albumin, and PTH concentrations were available. Patients with elevated creatinine levels, unclear ART or study ART, and therapy-naïve patients at the time of the blood sample were not enrolled in the study.

All laboratory results were obtained from single-time blood samples. Sex, date of birth, current ART regimen, date of blood sample, serum concentrations of creatinine, calcium, phosphate, albumin, and 25(OH)D, C-terminal telopeptides of collagen type 1 (*β*-CTx), and intact PTH were recorded. Albumin-corrected calcium levels (Ca_corr._) were calculated using Ca_corr._ (mmol/L) = Ca_total_ (mmol/L) − 0.025 · albumin (g/L) + 1 [7]. Quartiles were calculated based on the whole study populations.

Groups were compared for continuous and categorical variables using the Mann–Whitney *U* test and chi^2^-test, respectively. Spearman's rank method was used for correlations. In a multivariate median regression on PTH concentrations, Ca_corr._, sex, African ethnicity, age, serum creatinine, 25(OH)D, and the use of TDF, INI, PI, and NNRTI were included as covariates. The final model was built using a stepwise backwards approach, triggered by *P* values, including covariates with a *P* value <0.250 in univariate analysis.

Unless otherwise declared, data are reported as medians with the interquartile range (IQR). Results of regression were presented as regression coefficients with 95% confidence intervals. For visual analysis, fitted polynomial plots were used.

Statistical analysis was performed using STATA SE 13.1 software (Stata, College Station, TX, USA). A *P* value of <0.05 was considered statistically significant. This manuscript was written in accordance with guidelines for reporting observational studies (STROBE) [8].

The use of anonymized, routine clinical data does not require formal approval by local regulations.

## 3. Results

Altogether, 1,002 patients were included, of which 786 (78.4%) were male; median age was 48 (40–55) years. 564 patients (56.3%) had a TDF-containing ART regimen. Correlation of albumin-corrected calcium levels with PTH was weak (−0.129; *P* < 0.001) within the entire study population. While this weak correlation remained significant for PWLH using TDF (−0.130; *P*=0.002), it was not for PLWH without TDF-containing ART (−0.075; *P*=0.116). A detailed description can be found in Table 1.

**Figure 1:**
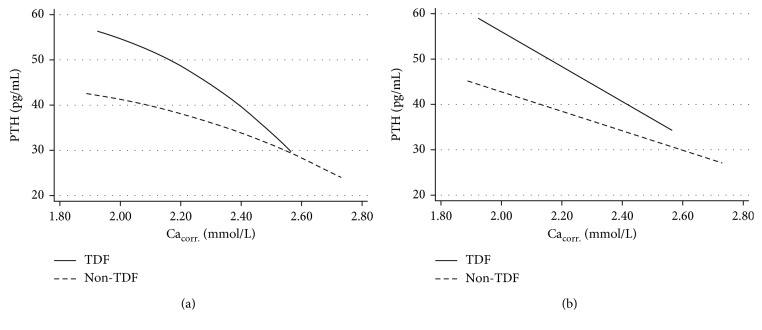
(a) Fitted fractional polynomial plot of predicted PTH concentration at a given albumin-corrected calcium concentration for patients on TDF- and non-TDF-containing ART. (b) Fitted linear plot of predicted PTH concentration at a given albumin-corrected calcium concentration for patients on TDF- and non-TDF-containing ART.

PTH concentrations were significantly higher throughout the entire range of Ca_corr._ when comparing patients with and without TDF-containing ART (Figures 1(a) and 1(b)) with 46.9 (33.0–64.7) pg/mL and 35.2 (26.4–55.4) pg/mL (*P*=0.001), 43.3 (30.8–59.8) pg/mL and 31.8 (22.3–49.6) pg/mL (*P* < 0.001), 46.1 (29.5–65.4) pg/mL and 33.4 (22.6–50.1) pg/mL (*P* < 0.001), and 37.8 (25.3–57.9) pg/mL and 33.8 (20.1–45.3) pg/mL (*P*=0.012) within the first, second, third, and fourth quartiles of Ca_corr._, respectively. PTH-Ca_corr._ ratio was 20.2 (13.7–28.4) and 15.1 (10.1–22.3) in patients with and without TDF-containing ART (*P* < 0.001).

After multivariate median regression, PTH concentration was significantly associated with Ca_corr._ (−32.2 (−49.8 to −14.8); *P* < 0.001), female sex (5.2 (1.2–9.2); *P*=0.010), 25(OH)D (−0.4 (−0.5 to −0.3); *P* < 0.001), and TDF-use (9.2 (6.0–12.5); *P* < 0.001).

**Figure 2:**
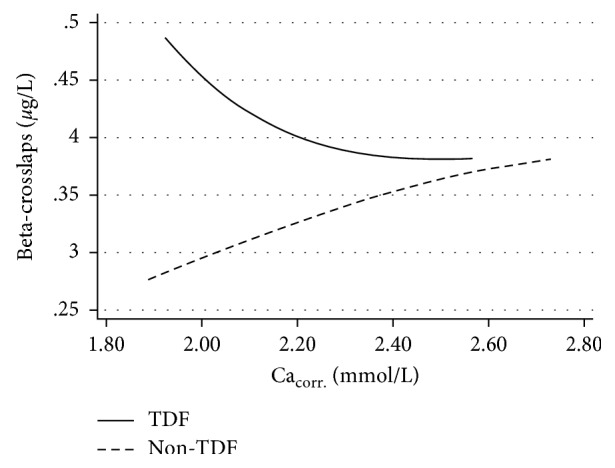
Fractional polynomial plots of predicted *β*-CTx levels for albumin-corrected calcium concentrations in patients with and without TDF-containing ART.

Ca_corr._ concentrations for patients with and without TDF-containing were stratified by 25(OH)D levels; detailed results can be found in Table 2. Correlations between Ca_corr._ and *β*-CTx were weak for PLWH on TDF (*ρ* = −0.079; *P*=0.061) as well as non-TDF-containing ART (*ρ* = 0.035; *P*=0.471) (Figure 2).

## 4. Discussion

In our study, higher levels of PTH were found in PLWH on TDF-containing ART compared to non-TDF-containing ART in general but, more importantly, also when adjusted for albumin-corrected calcium levels.

While higher levels of PTH have been described in people treated with TDF for chronic hepatitis B and HIV [1–3] as well as people taking TDF for PrEP [4] before, the underlying mechanisms have been poorly understood. Assuming ionized calcium levels to be the main regulator of PTH secretion, we therefore intended to investigate relations between PTH and Ca_corr._ levels in PLWH with TDF-containing and -free ART. Visualization of this association demonstrated higher levels of PTH throughout the whole continuum of Ca_corr._ levels when comparing PLWH with TDF-containing ART to those on non-TDF-containing regimens (Figure 1). This was substantiated by higher levels of PTH for all quartiles of Ca_corr._ levels in TDF-treated patients. These findings indicate that higher PTH concentrations in PWLH on TDF-containing ART are *not* associated with higher but rather *lower* levels of calcium (Figure 1). This cannot be explained by mere TDF-related inhibition of the CaSR that has been recently described [5] and seems to indicate other mechanisms being involved. Of particular interest in this context is the observation of a downregulation of Gnas gene products following TDF-exposure [9] that might translate into a degree of end-organ resistance to PTH and prevent compensation of hypocalcemia. Additionally, it has been shown that interaction with the osteoblast CaSR can lead to less-pronounced calcium-induced osteoblast maturation and differentiation with consecutive decreased mineralization [10]. It remains, however, unclear if the set-point variation of the calcium-PTH-axis in PLWH on TDF-containing ART is a “true” set-point variation or the result of an impaired capability of PTH to compensate low levels of calcium.

**Figure 3:**
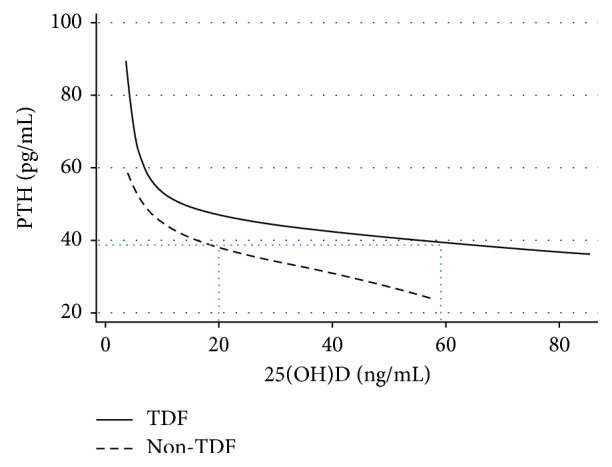
Fractional polynomial plots of predicted PTH concentration for serum 25(OH)D concentrations in patients with and without TDF-containing ART. Black dotted lines demonstrate that the degree of PTH suppression as found in non-TDF-treated patients at a threshold of 20 ng/mL (vitamin D deficiency) is only found at much higher 25(OH)D concentrations in PLWH on TDF-ART.

Interestingly, findings were similar for the association between PTH and 25(OH)D: while overall in good accordance with what has been described before [11], PTH levels tended to be higher in patients on TDF at each given 25(OH)D concentration throughout the whole range (Figure 3). This is a relevant finding given that one approach to defining normal ranges of 25(OH)D was the level of suppression of PTH [11, 12]: PTH levels in PLWH on non-TDF-containing ART at a 25(OH)D concentration of 20 ng/mL (a commonly used threshold to define 25(OH)D deficiency) were only found in much higher levels of 25(OH)D (about 58 ng/mL) in patients on TDF-ART. The “right-shift” of both curves might be seen as a hint for higher requirements in both, calcium and 25(OH)D in PLWH on TDF-ART, particularly but not exclusively with regard to bone health.

Combining our findings seem to indicate, that higher levels of both, Ca_corr._ (as high as 2.40 mmol/L) and 25(OH)D (as high as 60 ng/mL) might be needed to achieve a degree of PTH suppression that has previously been used to define optimal conditions in term of bone health. Adequate supply with vitamin D and calcium might therefore require a separate definition for PLWH on TDF-ART. This is supported by the observation of an additional, relevant PTH suppression following vitamin D supplementation in people using TDF seemingly regardless of baseline levels of 25(OH)D [13–15] and substantiated by finding of clinical trials [16–18].

Interestingly, hypocalcemia seems to translate differently into changes of turnover markers (BTM) in patients with and without TDF-containing therapy: while in TDF-patients, lower levels of Ca_corr._ were associated with increased BTM, in patients without TDF, lower levels of *β*-CTx were found, indicating low-bone turnover in patients with lower Ca_corr._ levels. This implies that low calcium levels in non-TDF-treated but not TDF-treated PLWH might have an inhibitory effect on bone turn over as demonstrated in distinct populations before [19].

One major limitation of our study is the retrospective design and the associated risk of unmeasured bias and the unavailability of data with potential influence including concentrations of serum magnesium levels, as well as intake of calcium and vitamin D. We do assume, however, that there is no systematic bias for any of those parameters towards any of the groups. Another limitation is the lower number of patients in the upper end of the ranges of 25(OH)D and Ca_corr._ with consequently possibly not very robust point estimates. Finally, and probably most importantly, the meaning of elevated PTH in patients on TDF is an ongoing matter of debate. While intuitive, it has never been clearly demonstrated that elevated PTH levels are associated with higher risk of loss in bone mineral density (BMD); recent work even suggested an inverse relationship in PLWH with PTH concentrations being associated with increased decline in BMD of cortical bone in longitudinal observation [20]. Last, preanalytic issues for both, PTH as well as BTM, play a crucial role concerning the laboratory results. Due to instability of PTH in delayed processing of blood samples as well as suppressive effect of nutrition on levels of *β*-CTx, we might have a systemic trend for “false low” results. They should, however, be randomly assigned to both groups and therefore not lead to a systemic error in our analyses.

The generalizability of our data is limited by the racial influence on vitamin D and PTH metabolism in PLWH as demonstrated before [21]. This might also be true for other environmental factors such as geographical location. We do however assume that differences as described in our study will also be found in other study populations, while only their extend might differ. It is furthermore unclear whether our data can be generalized to non-HIV-infected people using TDF, as HIV-associated alterations of PTH secretion have been acknowledged [13, 22] and osteoimmunologic aspects might be different in PLWH compared to non-HIV-infected people.

## 5. Conclusion

TDF-exposure seems to induce a complex disturbance of the calcium-parathyroid hormone-vitamin D axis in PLWH with characteristics of primary and secondary hyperparathyroidism, as well as pseudohypoparathyroidism. Optimal conditions for bone health might therefore be different in PLWH with TDF-ART compared to those on TDF-free regimens (possibly including TAF), indicated by PTH suppression at higher levels of both, calcium and 25(OH)D. Given the increasing body of evidence of low calcium levels being associated with favorable cardiovascular survival and the recent data about TDF being negatively associated with incident heart failure [23], we do not want to suggest aggressive supplementation strategies of calcium and vitamin D in PLWH on TDF therapies but rather encourage further research into cardiovascular effects of TDF and the question of whether or not this can also be assumed for TAF-based regimens, as well as possible supplementation strategies.

## Figures and Tables

**Table 1:** Patient characteristics of groups with and without TDF-containing ART.

	Unit	TDF (*N*=564)	Non-TDF (*N*=438)	*P* value
Age	Years (IQR)	47 (38–53)	50 (42–58)	**<0.001**
Male	*N* (%)	429 (76.1)	357 (81.5)	**0.038**
African ethnicity	*N* (%)	91 (16.1)	33 (7.5)	**<0.001**
Missing data	*N* (%)	1 (0.2)	0 (0.0)	
Creatinine (*S*)	mg/dL (IQR)	0.95 (0.82–1.07)	1.00 (0.86–1.11)	**<0.001**
25(OH)D (*S*)	ng/mL (IQR)	23.0 (14.9–31.1)	23.6 (16.5–30.4)	0.366
25(OH)D < 20 ng/mL	*N* (%)	230 (40.8)	165 (37.7)	0.998
Total calcium (*S*)	mmol/L (IQR)	2.22 (2.16–2.28)	2.25 (2.18–2.31)	**<0.001**
Albumin-corrected calcium	mmol/L (IQR)	2.20 (2.14–2.26)	2.22 (2.17–2.28)	**<0.001**
Albumin-corrected calcium < 2.12 mmol/L	*N* (%)	96 (17.0)	43 (9.8)	**0.001**
Phosphate	mg/dL (IQR)	3.4 (3.0–3.8)	3.4 (3.0–3.8)	0.392
PTH (*S*)	pg/mL (IQR)	44.3 (29.9–61.9)	33.7 (22.5–49.3)	**<0.001**
Elevated PTH	*N* (%)	121 (21.5)	46 (10.5)	**<0.001**
C-terminal telopeptides of collagen type 1 (*S*)	*µ*g/L (IQR)	0.38 (0.25–0.52)	0.28 (0.20–0.42)	**<0.001**
Elevated C-terminal telopeptides of collagen type 1	*N* (%)	49 (8.7)	25 (5.7)	0.074
Alkaline phosphatase (*S*)	U/L (IQR)	83 (68–101)	74 (61–93)	**<0.001**

**Table 2:** Concentrations of albumin-corrected calcium levels in PLWH with and without TDF-containing ART stratified by 25(OH)D concentrations.

		25(OH)D concentrations		
		<20 ng/mL	≥20–40 ng/mL	≥40–60 ng/mL
Ca_corr._ (mmol/L) median (IQR)	TDF (*N*=564)	2.21 (2.15–2.28)	2.19 (2.13–2.25)	2.18 (2.14–2.24)
	Non-TDF (*N*=438)	2.22 (2.17–2.28)	2.21 (2.17–2.28)	2.21 (2.16–2.28)
*P* value		0.192	0.001	0.296

## Data Availability

The data used to support the findings of this study are available from the corresponding author upon request.

## Abbreviations

ABC: Abacavir
ART: Antiretroviral therapy
Ca_corr._: Albumin-corrected calcium concentration
CaSR: Calcium-sensing receptor
INI: Integrase inhibitor
IQR: Interquartile range
NNRTI: Nonnucleoside reverse transcriptase inhibitor
NRTI: Nucleoside/nucleotide reverse transcriptase inhibitor
TAF: Tenofovir alafenamide
TDF: Tenofovir disoproxil fumarate
PI: Protease inhibitor
PrEP: Pre-exposure prophylaxis (for HIV)
PTH: Parathyroid hormone
PWLH: People living with HIV
25(OH) D: 25-hydroxy vitamin D
*β*-CTx: C-terminal telopeptides of collagen type 1.
